# Rapamycin Inhibited Pyroptosis and Reduced the Release of IL-1*β* and IL-18 in the Septic Response

**DOI:** 10.1155/2020/5960375

**Published:** 2020-08-08

**Authors:** Luo Zhuo, Xiaobing Chen, Yan Sun, Yanli Wang, Yuanfeng Shi, Lin Bu, Wei Xia, Jiayan Han, Dongmei Chen, Xiaomin Li

**Affiliations:** ^1^Department of Emergency Medicine, The Affiliated Lianyungang Hospital of Xuzhou Medical University, No. 182 North Tongguan Road, Lianyungang 222002, China; ^2^Department of Emergency Medicine, The Clinical Medical School of Nanjing Medical University Affiliated Hospital of Lianyungang First People's Hospital, No. 182 North Tongguan Road, Lianyungang 222002, China; ^3^Department of Critical Care Medicine, Xuzhou Medical University Affiliated Hospital, No. 99 West Huaihai Road, Xuzhou 221000, China; ^4^Department of Intensive Care Unit, The Affiliated Wuxi People's Hospital of Nanjing Medical University, No. 299 Qingyang Road, Wuxi 214000, China

## Abstract

Pyroptosis, an inflammatory form of programmed cell death, is the initiating event of sepsis and results in immune imbalance by releasing IL-1*β* and IL-18 in the early stages. Studies show that enhancing autophagy via genetic manipulation can inhibit pyroptosis and prolong the survival of a sepsis animal model, indicating a possible therapeutic strategy against sepsis. However, almost no study so far has achieved pyroptosis inhibition via pharmacological autophagy induction in a sepsis disease model. To this end, we established an *in vitro* sepsis model by stimulating primary human umbilical vein endothelial cells (HUVECs) with lipopolysaccharide (LPS), and analyzed the effect of the autophagy agonist rapamycin (RAPA) on pyroptosis. Phorbol 12-myristate 13-acetate- (PMA-) activated human THP-1 cells were used as the positive control. LPS significantly increased the levels of the pyroptotic protein Gasdermin D (GSDMD), cysteinyl aspartate-specific proteinase 1 (caspase-1), secreted LDH, IL-1*β*, and IL-18. RAPA treatment downregulated the above factors and enhanced autophagy in the LPS-stimulated HUVECs and THP-1 cells. This study shows that RAPA abrogates LPS-mediated increase in IL-1*β* and IL-18 by inhibiting pyroptosis and enhancing autophagy.

## 1. Introduction

**Figure 1:**
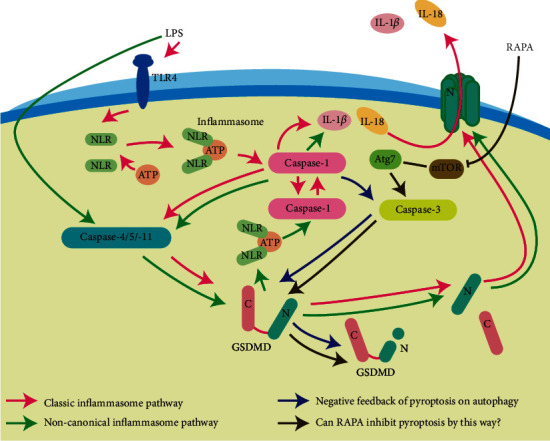
The red arrow shows the classical pathway of LPS-mediated activation of TLR4 that further leads to pyroptosis and releases of IL-1*β* and IL-18. The green arrow shows the noncanonical pathway through which LPS directly activates caspase-4/5/11 without TLR4. The blue arrow shows the negative feedback of pyroptosis on autophagy wherein caspase-1-mediated activation of caspase-3 leads to enhanced autophagy and inactivation of GSDMD. The brown arrow shows RAPA-mediated deactivation of the inhibitory effect of mTOR on Atg7 that causes Atg7 to activate caspase-3. We surmise that RAPA is also more destructive to GSDMD and inhibits pyroptosis.

Pyroptosis is an inflammatory form of programmed cell death that is triggered following infection with intracellular pathogens and may contribute to the innate immune response [1, 2]. At the molecular level, it begins with the recognition of the pathogen-associated molecular patterns (PAMP) or damage-associated molecular patterns (DAMP) by the pattern recognition receptor (PRR) on the host immune cells, which then induces the inflammasome pathway [3]. The latter cleaves caspase-1, which in turn cleaves the downstream caspases (caspase-4/5 in humans and 11 in mice), as well as the prointerleukin-1 beta (IL-*β*) and IL-18 into their active forms. This eventually triggers the caspase-mediated pyroptotic cascade and releases high levels of the inflammatory cytokines IL-1*β* and IL-18 [4] (Figure 1, the red arrow).

Sepsis is a potentially fatal medical condition that results from the body's response to bacterial infection and can lead to multiple organ failure, septic shock, and even death [5, 6]. Since it is usually diagnosed at the later stages, the treatment is limited to supportive care for the organ dysfunction [7]. Therefore, it is vital to understand the mechanisms underlying the sequence of sepsis, in order to prevent its onset as well as rapidly diagnose the condition and initiate therapy at the earliest.

Studies show that pyroptosis is the trigger of the cytokine storm seen at the beginning of sepsis [4, 8]. In addition, knockout of the gene encoding the pyroptotic protein Gasdermin D (GSDMD) and overexpression of autophagy-related proteins can effectively inhibit pyroptosis and prolong the survival of a sepsis animal model [4, 9–11].

Kim et al. found that microglial cells infected with *Streptococcus pneumoniae* overexpressed the autophagy-related proteins and exhibited low levels of pyroptosis, while inhibition of autophagy in the infected cells upregulated caspase-1 and increased IL-18 release [12]. Consistent with this, Pu et al. showed that inhibition of the autophagy-related protein Atg7 led to uncontrolled activation of inflammasomes in mice infected with *Pseudomonas aeruginosa* [13]. Treatment with the autophagy agonist rapamycin (RAPA) abrogated the effects of Atg7 inhibition [14].

LPS is a PAMP that is recognized by the Toll-like receptor 4 (TLR4), which activates the downstream pyroptotic pathway [15]. LPS-stimulated cells are an established *in vitro* model of sepsis and simulate the first 24 hours [16]. Therefore, we studied the occurrence of pyroptosis and autophagy as well as the release of IL-1*β* and IL-18 after LPS stimulation of HUVECs and macrophages with RAPA, in order to understand whether the latter can be used in the earliest stages of sepsis to block inflammatory cytokine storm.

## 2. Materials and Methods

### 2.1. Cell Culture and Treatment

Primary human umbilical veil endothelial cells (HUVECs) were obtained from the Sciencell Research Laboratories, USA, and cultured in the Endothelial Cell Medium (ECM, Sciencell Research Laboratories). The cells from the first 5 passages were seeded in 6-well or 96-well and cultured with 10 *μ*g/ml LPS (Sigma, USA) for varying durations, in the presence/absence of 5 *μ*M RAPA (MedChemExpress, USA) for 24 h. The cells were harvested, and supernatants were also collected for the downstream assays.

Human THP-1cells were obtained from the Chinese Academy of Sciences cell bank and cultured in RPMI 1640 cell medium (Nanjing KeyGen, China) supplemented with 10% FBS. Differentiation into macrophages was induced by a 48 h treatment with 100 ng/ml PMA (MedChemExpress, USA) after 5 passages [17]. The macrophages were washed thrice with PBS and cultured in fresh medium for 24 h with 1 *μ*g/ml LPS (Sigma, USA) for varying durations in the presence/absence of 5 *μ*M RAPA (MedChemExpress, USA).

### 2.2. Western Blotting

The suitably treated HUVECs and macrophages were homogenized in the RIPA lysis buffer containing 1% phenylmethanesulfonyl fluoride (PMSF) for extracting total cellular protein. The cell lysates were separated by sodium dodecyl sulfate polyacrylamide gel electrophoresis (SDS-PAGE) and transferred to polyvinylidene fluoride (PVDF) membranes (Millipore, 0.22 *μ*m for LC3 and 0.45 *μ*m for others, USA). After blocking with 5% milk powder for 90 minutes at room temperature, the membranes were incubated overnight with primary antibodies against caspase-1 (Abcam, UK), GSDMD (Abcam) LC3 (Cell Signaling Technology, USA), and GAPDH (ABclonal, USA) at 4°C, followed by incubation with peroxidase-conjugated secondary antibodies. The positive bands were detected using an electrochemiluminescence (ECL) reagent according to the manufacturer's instructions.

### 2.3. LDH Release Assay

The lysates and supernatants of the LPS and rapamycin-treated HUVECs and macrophages (200 *μ*l per sample) were dispensed into each well of a 96-well assay plate. The chromogenic reagent provided in the LDH assay kit was then added, and the luminescence signals were measured using a microplate spectrophotometer. The relative amount of LDH was calculated according to the following formula: LDH relative release amount (%) = (absorbance of treated sample − absorbance of control hole of sample)/(absorbance of maximum enzyme activity of cells − absorbance of control hole of sample)∗100.

### 2.4. Electron Microscopy

The HUVECs were fixed overnight with 2.5% glutaraldehyde solution at 4°C, washed thrice with PBS, fixed further with osmium for 2.5 hours, and rinsed thrice with PBS. The fixed cells were embedded in resin blocks through sequential ethanol gradient dehydration, ethanol acetone replacement, and acetone resin replacement. Ultrathin sections were cut, stained with uranium acetate and lead citrate, and observed using an electron microscope (JEM-1200EX, Japan Electron Optics Laboratory, Japan).

### 2.5. ELISA

The secreted levels of IL-18 in the HUVECs and macrophages supernatants were measured using a Human IL-18 ELISA kit (ABclonal, USA) according to the manufacturer's instructions.

The levels of secreted IL-1*β* in the macrophage culture supernatants were measured using a Human IL-1*β* ELISA kit (ABclonal, USA) according to the manufacturer's instructions. Since the IL-1*β* levels in the HUVECs supernatants were lower than the range of the kit, it was not considered in our experiments.

### 2.6. Statistical Analysis

GraphPad Prism 7.0 (La Jolla, USA) was used for statistical analysis and graph plotting. One-way analysis of variance (ANOVA) was used to compare the data, and *P* value < 0.05 was considered statistically significant. Every experiment was performed at least thrice.

## 3. Results

### 3.1. LPS Induced Pyroptosis and Triggered IL-1*β* and IL-18 Release

**Figure 2:**
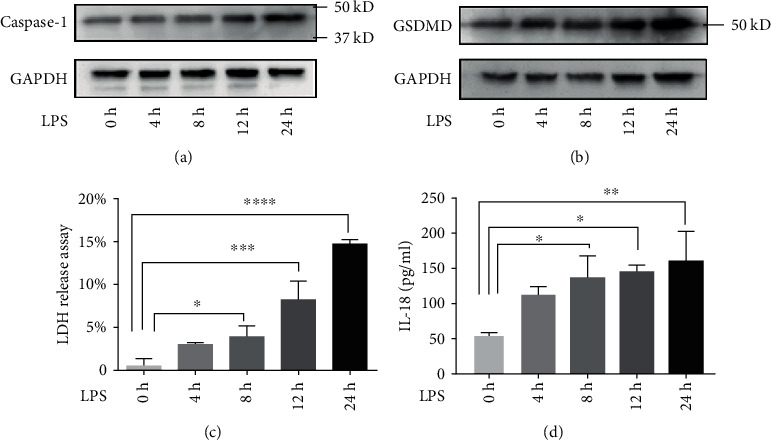
Establishment of LPS-stimulated HUVECs sepsis model. (a, b) Immunoblots showing caspase-1 and GSDMD levels in the HUVECs treated with LPS for 0, 4, 8, 12, and 24 h. (c) LDH content in supernatant at the different time points. (d) IL-18 secretion in the supernatant following LPS stimulation. ^∗^*P* < 0.05, ^∗∗^*P* < 0.01, ^∗∗∗^*P* < 0.001, and ^∗∗∗∗^*P* < 0.0001.

**Figure 3:**
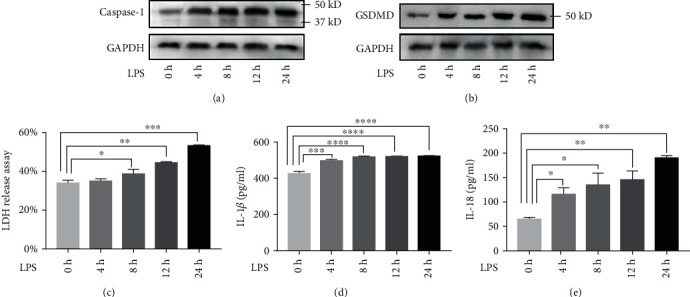
Establishment of LPS-stimulated THP-1 sepsis model. (a, b) Immunoblots showing caspase-1 and GSDMD levels in the THP-1 treated with LPS for 0, 4, 8, 12, and 24 h. (c). LDH content in the supernatant at the different time points. (d, e) IL-1*β* and IL-18 secretion in the supernatant following LPS stimulation. ^∗^*P* < 0.05, ^∗∗^*P* < 0.01, ^∗∗∗^*P* < 0.001, and ^∗∗∗∗^*P* < 0.0001.

To explore the role of RAPA intervention in pyroptosis of septic response, two sepsis models were established. HUVECs were cultured with LPS, while human THP-1 cells were induced to differentiate into macrophages, then were cultured with LPS. A significant time-dependent increase of IL-18 in supernatant was found in both models (*P* < 0.05, Figure 2(d) and 3(e)) and IL-1*β* increased in THP-1 model (*P* < 0.001, Figure 3(d)). The LDH content in the supernatant increased progressively with time which was indicative of membrane disintegration (Figure 2(c) and 3(c)). Further, western blotting were used to assess pyroptosis-related protein. As shown in Figures, LPS treatment stimulated the upregulation of caspase-1 (Figure 2(a) and 3(a)) and GSDMD (Figure 2(b) and 3(b)) expression. These indicators all strongly suggested pyroptosis occurred in LPS-stimulated sepsis models.

### 3.2. RAPA Has No Effect on Pyroptosis and IL-1*β* and IL-18 Release

**Figure 4:**
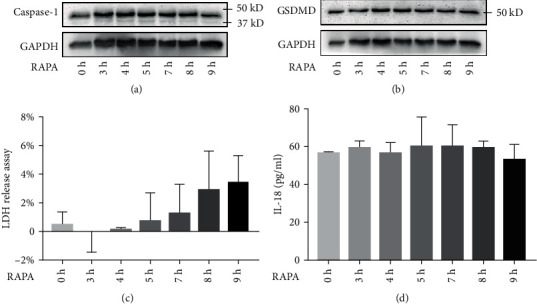
Effect of RAPA on HUVECs. (a, b) Immunoblot showing intracellular caspase-1 and GSDMD levels. (c) LDH and (d) IL-18 levels in the supernatant.

**Figure 5:**
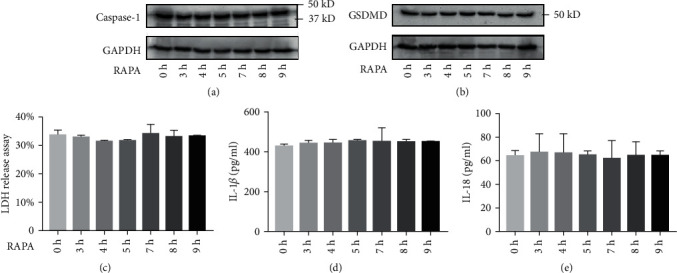
Effect of RAPA on THP-1. (a, b) Immunoblot showing intracellular caspase-1 and GSDMD levels. (c) LDH, (d) IL-1*β*, and (e) IL-18 levels in the supernatant.

To determine the effect of RAPA on pyroptosis, the HUVECs and macrophages were treated with RAPA alone. As shown in Figures 4 and 5, there were no significant differences in the expression of pyrogen-related proteins, the content of LDH in the supernatant, and the release of IL-18 and IL-1*β* in the experimental group compared to the control group (*P* > 0.05). RAPA had no significant effect on the levels of the pyroptosis indicators.

### 3.3. RAPA-Mediated Autophagy Abrogated the Effects of LPS

**Figure 6:**
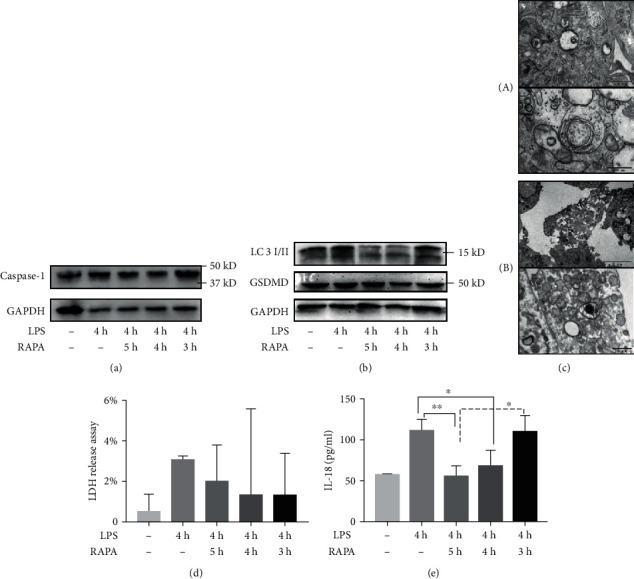
RAPA abrogates the effect of 4 h LPS stimulation in HUVECs. (a, b) Immunoblot showing the levels of caspase-1, GSDMD, LC3II, and LC3I. (c) Electron microscopy images showing autophagosomes in the (A) LPS- and (B) LPS+RAPA-treated HUVECs. (d) Relative concentration of LDH in the supernatant. (e) IL-18 content released into the supernatant. ^∗^*P* < 0.05 and ^∗∗^*P* < 0.01.

**Figure 7:**
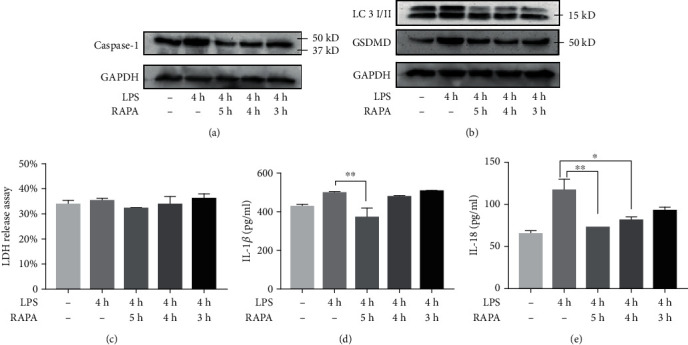
RAPA abrogates the effect of 4 h LPS stimulation in THP-1. (a, b) Immunoblot showing the levels of caspase-1, GSDMD, LC3II, and LC3I. (c) Relative concentration of LDH in the supernatant. (d) IL-1*β* and (e) IL-18 content released into the supernatant. ^∗^*P* < 0.05 and ^∗∗^*P* < 0.01.

**Figure 8:**
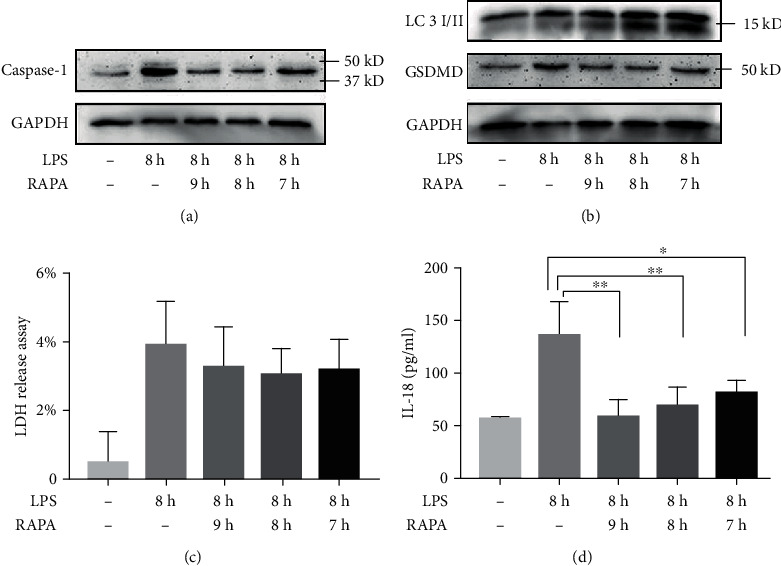
RAPA abrogates the effect of 8 h LPS stimulation in HUVECs. (a, b) Immunoblot showing the levels of caspase-1, GSDMD, LC3II, and LC3I. (c) Relative concentration of LDH in the supernatant. (d) IL-18 content released into the supernatant. ^∗^*P* < 0.05 and ^∗∗^*P* < 0.01.

**Figure 9:**
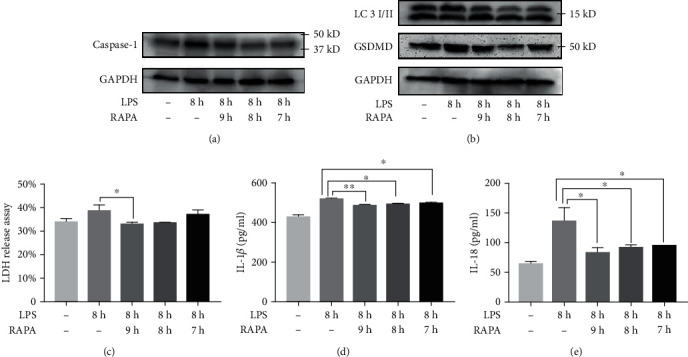
RAPA abrogates the effect of 8 h LPS stimulation in THP-1. (a, b) Immunoblot showing the levels of caspase-1, GSDMD, LC3II, and LC3I. (c) Relative concentration of LDH in the supernatant. (d) IL-1*β* and (e) IL-18 content released into the supernatant. ^∗^*P* < 0.05 and ^∗∗^*P* < 0.01.

RAPA was found to be cytoprotective against LPS-induced pyroptosis (Figures.  6–9). Based on the existing studies and the results of the LPS-stimulated sepsis model experiments, two time points, 4 h and 8 h, were selected as the experimental time points [13]. The HUVECs and macrophages were treated with RAPA either 1 h before (pre-intervention group), simultaneously, or 1 h after (postintervention group) a 4 h or 8 h LPS stimulation.

The synthesis of the pyroptotic proteins caspase-1 and GSDMD protein was decreased in the preintervention and intervention groups of HUVECs and macrophages in the 4 h modeling group compared to the control group (Figures 6(a), 6(b), 7(a), and 7(b)). In addition, extracellular LDH content and secretion in IL-1*β* and IL-18 were significantly decreased (Figure 6(d), 6(e), 7(c), and 7(e); *P* < 0.05). The cells showed increased the LC3II to LC3I ratio after RAPA intervention (Figures 6(b) and 7(b)), and HUVEC electron microscopy results also showed a greater increase of autophagosomes and lysosomes compared to the LPS-stimulated HUVECs which unexposed to RAPA (Figure 6(c)). Interestingly, some RAPA-treated cells also showed the characteristic membrane blebbing of apoptosis (Figure 6(c)).

We similarly observe that RAPA downregulated the pyroptotic markers, IL-1*β*, IL-18, and LDH secretion, and increased LC3II to LC3I ratio (Figure 8(a)–8(c) and Figure 9(a)–9(d)) in HUVECs and macrophages stimulated with LPS for 8 h. Some of these differences were also statistically significant relative to the control (*P* > 0.05).

Notably, both RAPA postintervention subgroups of HUVECs and macrophages in the group of LPS-stimulated cells for 4 h have no significant difference in caspase-1 and GSDMD expression, LDH content, and release of IL-1*β* and IL-18 compared with the LPS-stimulated group at the same time (Figure 6 and 7; *P* > 0.05).

## 4. Discussion

It is demonstrated that RAPA can reduce the pyroptosis in various cell models [18–20] and inhibition of pyroptosis prolong the survival of a sepsis animal model [4, 9–11]. In the present study, we found that RAPA inhibited pyroptosis and reduced the septic response in both LPS-induced HUVECs and PMA+LPS-activated human THP-1 cell models of sepsis. RAPA terminated the excessive inflammatory response by maintaining the integrity of the membrane of sepsis cell model and reducing the release of IL-1*β* and IL-18 from these immune cells, which are the pivotal inflammation mediators in sepsis.

As an important mode of cell death in early sepsis, pyroptosis is likely to be a major cause of the immune storm because of its concomitant release of the proinflammatory factors IL-1*β* and IL-18 [21]. The secretion of IL-18 by endothelial cells, mononuclear macrophages, and epithelial cells is increased during bacterial infection, which promotes IFN-*γ* release from the T lymphocytes or NK cells. Both IFN-*γ* and IL-18 stimulate macrophages to release large amounts of IL-1*β*, further aggravating the inflammatory stress response [22, 23]. Endothelial cells line the blood vessels of various organs and undergo pathophysiological changes during the course of sepsis [24, 25]. PMA-activated THP-1 cells can replace human macrophages in *in vitro* tests [26]. The *in vitro* models of sepsis were successfully established via stimulating HUVEC and macrophages by LPS for 24 hours, which showed pyroptosis of HUVECs and macrophages with the release of proinflammatory factors IL-1*β* and IL-18.

RAPA is a classical mTOR inhibitor that promotes caspase-3 activation by regulating downstream autophagy-associated proteins via the PI3K/AKT/mTOR pathway [27]. Caspase-3 inactivates the GSDMD N-terminal domain by nonspecific cutting during autophagy, which is a negative feedback regulation to pyroptosis [10]. In this study, RAPA was effective in reducing release of IL-1*β* and IL-18 *in vitro* septic model cells, which perhaps further inhibit the inflammatory response. This massive infiltration of inflammatory cells and the cytokine storm are responsible for the rapid progression and recalcitrance at the beginning of sepsis [28]. In our experiments, we observed that RAPA reduced the expression of pyroptotic proteins, caspase-1 and GSDMD, decreased the release of LDH release, decreased IL-1*β* and IL-18, and increased LC3II to LC3I ratio. This suggests that RAPA can inhibit LPS-induced cellular pyroptosis, protect cell membrane integrity, and reduce the release of inflammatory factors IL-1*β* and IL-18 into the extracellular compartment by enhancing autophagy. Due to the limitation of method, little effect on LDH secretion was observed but with a trend of decreased membrane destruction. If the direct quantitative experiment was conducted, statistical significance might have been found. Because of the pathophysiological changes in sepsis, the presence of apoptosis was also observed in our electron microscope results. It is consistent with a previous study that pyroptosis, apoptosis, and necrosis are present in sepsis and are interconnected [1].

Collectively, the study showed that RAPA inhibited pyroptosis and protected immune cells from excessive inflammation in the septic response. These data suggest that RAPA enhanced autophagy, reduced the release of proinflammatory factor IL-1*β* and IL-18, and blocked inflammatory cytokine storm, which is a potential mechanism that RAPA could mitigate the overreaction of the immune system in course of sepsis.

## Data Availability

The generated or analyzed data used to support the findings of this study are included within the article.
